# Dedifferentiated retroperitoneal large liposarcoma and laparoscopic treatment: Is it possible and safe? The first literature case report

**DOI:** 10.1016/j.ijscr.2019.03.023

**Published:** 2019-03-26

**Authors:** Antonino Agrusa, Giuseppe Di Buono, Salvatore Buscemi, Brenda Randisi, Leonardo Gulotta, Vincenzo Sorce, Giuseppe Badalamenti, Domenico Albano, Massimo Galia, Giorgio Romano, Gaspare Gulotta

**Affiliations:** a Department of Surgical, Oncological and Oral Sciences, Section of General and Urgent Surgery, University of Palermo, Italy; b Department of Surgical, Oncological and Oral Sciences, Section of Medical Oncology, University of Palermo, Italy; c Unit of Diagnostic and Interventional Radiology, IRCCS Istituto Ortopedico Galeazzi, Milan, Italy; d Department of Radiology, University of Palermo, Palermo, Italy

**Keywords:** Dedifferentiated liposarcoma, Retroperitoneal liposarcoma, 3D laparoscopic surgery

## Abstract

**Introduction:**

Soft tissue sarcomas are rare neoplasms often characterized by local invasiveness and distant metastasis with poor prognosis for affected patients. Among the most frequent sarcomas we find well-differentiated and dedifferentiated liposarcomas characterized by a better survival compared to the other histological types. When it is possible the only curative treatment for these neoplasms is surgical resection.

**Case report:**

We report a case of a 62-year-old caucasian woman with CT abdominal scan that demonstrated a voluminous solid oval mass in the left perirenal space with dislocation of the kidney and in continuity with the anterior renal fascia. After a percutaneous CT-guided biopsy of the mass the histopathological diagnosis was a dedifferentiated retroperitoneal liposarcoma. We performed a laparoscopic surgical resection of the retroperitoneal mass in block with kidney and left adrenal gland.

**Discussion:**

When possible, surgical resection with adequate margins represents the only curative therapeutic option for this pathology. Only a few papers are available in literature which take into consideration the possibility of laparoscopic approach for retroperitoneal masses with better vision of surgical field, reduction of post-operative pain and better cosmesis.

**Conclusion:**

A retroperitoneal mass represents a serious diagnostic challenge. The choice of the best surgical procedure can benefit to the patient prognosis. To our opinion laparoscopy can be a safe and successful treatment and it can represent a valid alternative to open surgery. However, we have no randomized controlled trials that compare laparoscopic versus open resection for retroperitoneal liposarcomas.

## Highlights

• We describe a case report of large retroperitoneal dedifferentiated liposarcoma totally treated by laparoscopic surgery.
• In literature we found few cases of laparoscopic treatment only for Well-Differentiated liposarcoma.
• To our knowledge this is the first description of Dedifferentiated liposarcoma completely treated with laparoscopic technique.
• Literature review was performed to identify outcomes and advantages of laparoscopic approach for.

## 1 Introduction

Soft tissue sarcomas are rare neoplasms (1% of all solid tumors in adults) often characterized by local invasiveness and distant metastasis with poor prognosis for affected patients [1]. 15–20% of these tumors are approximately located in the retroperitoneum. Among the most frequent sarcomas we find well-differentiated (WD) and dedifferentiated (DD) liposarcomas characterized by a better survival compared to the others histotypes [2,3]. If possible the only curative treatment for these neoplasms is surgical resection. Although many authors always prefer an open approach for surgical procedures in literature we found some cases of laparoscopic treatment only for WD liposarcoma [4]. To our knowledge this is the first description of DD liposarcoma completely treated with laparoscopic technique and it is reported in line with the SCARE criteria [5,6].

## 2 Case report

**Fig. 1:**
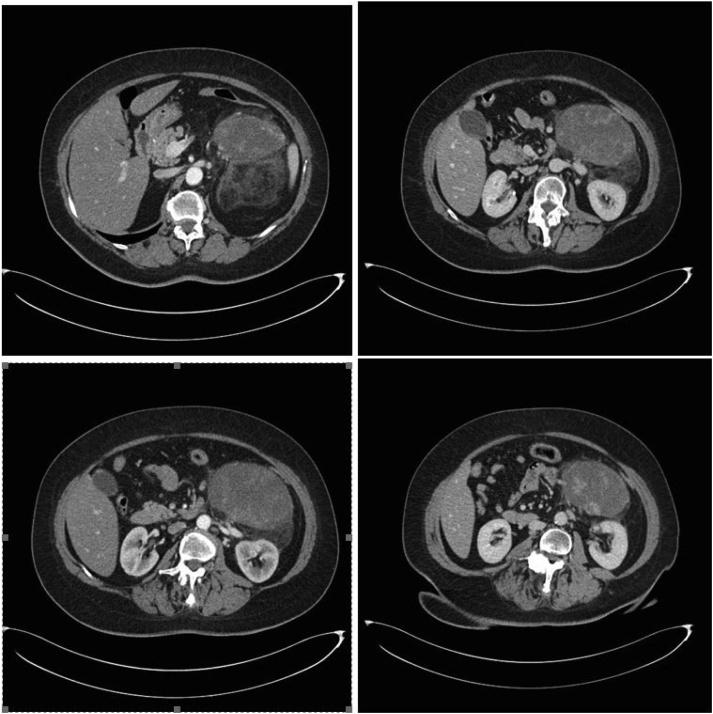
CT abdominal scan showed a voluminous solid oval mass (11.2 cm × 7.5 cm × 12 cm) in the left perirenal space with dislocation of the kidney and in continuity with the anterior renal fascia. After iodinated-contrast we observed a progressive, inhomogeneous enhancement of the lesion with peripheral vascularization.

**Fig. 2:**
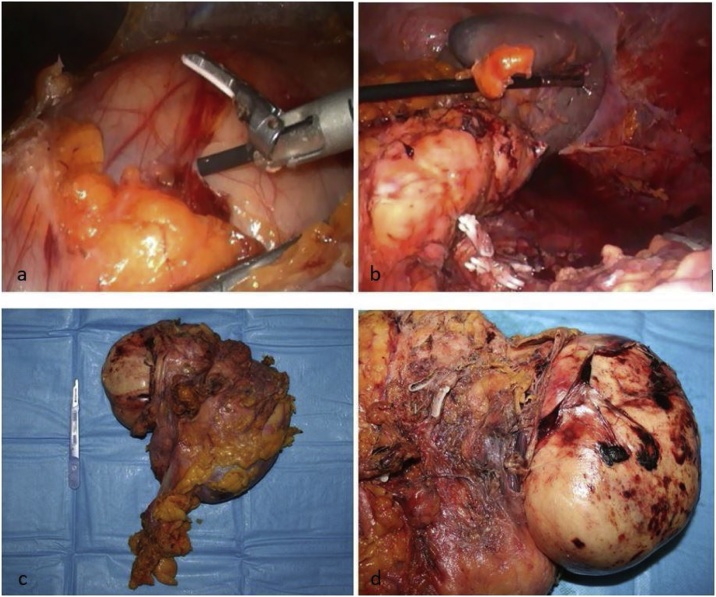
a) Laparoscopic dissection of adiposes cleavage planes with the left colon and the others surrounding peritoneal organs; b) retroperitoneal space after surgical resection: we can identify spleen and preserved pancreatic tail, clipped left renal vein and artery; c) surgical specimen with in block resection of left kidney, adrenal gland and DD liposarcoma; d) DD liposarcoma appeared oval, 13 × 11 × 9 cm of size, with a smooth surface and regular margins.

A 62-year-old caucasian woman came to the emergency room with fever for a month and pain in the left upper quadrants of the abdomen and lower left back pain. She had no other systemic symptoms or comorbidity. Routine blood tests were in the normal range with no evidence of infectious diseases. On clinical examination there were no signs of peritonitis but we found a palpable large mass in left flank. We performed a CT abdominal scan that demonstrated a voluminous solid oval mass (11.2 cm × 7.5 cm × 12 cm) in the left perirenal space with dislocation of the kidney and in continuity with the anterior renal fascia. After iodinated-contrast we observed a progressive, inhomogeneous enhancement of the lesion with peripheral vascularization (Fig. 1) [7,8]. After a percutaneous CT-guided biopsy of the mass the histopathological diagnosis was a dedifferentiated retroperitoneal liposarcoma. Considering the site of the neoplasm in left renal loggia and the absence of others repetitive local or distant lesions our tumor board decided for surgical resection of the mass in block with kidney and left adrenal gland. We chose the possibility of laparoscopic approach with conversion to open surgery in case of muscle infiltration or vascular invasion. After preoperative clinical study [9] the surgical procedure was carried out with a transperitoneal approach with the patient in right lateral decubitus position to obtain a large surgical field with well known anatomic landmarks and possibility of exploration of peritoneal organs. We used a laparoscopic 3D vision system [10,11] with three trocars in the left subcostal region, but during surgical procedure we positioned another 5-mm trocar for spleen retraction. We performed an adequate mobilization of the splenic-pancreatic block in order to identify infiltration of peritumor tissues. We found a well-capsulated mass. The dissection was done with Harmonic scalpel™ (Ethicon Endo Surgery INC – Johnson & Johnson, NJ, USA) from up-to-down until the left renal artery and vein and the ureter were clipped and divided with the aim to perform an in block resection of left adrenal gland, kidney and DD liposarcoma with safety margins adequate for neoplasm resection (Fig. 2a and b). At the end of procedure surgical specimens were positioned in endo-bag and we used Tesseel™ (Baxter International Inc - Deerfield, Illinois, USA) for repositioning splenic-pancreatic block. We left a drain in retroperitoneal space and we did a sovrapubic minilaparotomy for extraction of resected mass [[12], [13], [14], [15], [16], [17], [18]]. Macroscopically the mass appeared oval, 13 × 11 × 9 cm of size, with a smooth surface coated with a greyish capsule and with regular margins (Fig. 2c and d). The morphological and immunophenotypic characteristics (MDM2 +, vimentin +, S100 +, SMA+, pancytokeratin −, desmin −, CD34 −, ki67 = 20%) confirmed the diagnosis of dedifferentiated liposarcoma (according to WHO 2013). The kidney, the perirenal adipose tissue, the ureter, the adrenal gland and the retroperitoneal lymph nodes appeared to be free from neoplastic infiltration. The patient was discharged from our hospital a week later. On the last follow-up control (about 12 months later) she was in good general clinical condition and without postoperative radiologic evidence of tumor recurrence.

## 3 Discussion

Sarcomas are malignant tumors of soft tissues. They derive from connective tissue [1] and represent the 1% of all malignant tumors [2]. Retroperitoneal liposarcoma accounts for 35–40% of all retroperitoneal sarcomas and it generally occurs in the sixth and seventh decades of life [[19], [20], [21]]. Often the patients are asymptomatic or they feel non-specific symptoms due to compression of the surrounding organs such as lower back pain, fever or early satiety [22,23]. The absence of symptoms determines the progressive growth of these tumors that can reach even large dimensions as in our case report. Frequently these tumors remain unknown [24,25] or present themselves as incidentaloma. Liposarcomas histologically range from well-differentiated to dedifferentiated tumors that are more aggressively and can metastasize [2,23] with a 5-years survival rate between 44 and 53% [26]. When it is possible surgical resection with disease free margins represents the only curative therapeutic option [23,27,28]. In particular with regarding to the surgical treatment this is usually performed with open technique. Only few case reports or case series available in literature take into consideration the possibility of laparoscopic approach. All these articles treat WD liposarcomas. To our knowledge this is the first reported case of totally laparoscopic treatment of a DD liposarcoma. Nomura et al. describe the case of a WD retroperitoneal liposarcoma with laparoscopic technique and consider only four others liposarcomas treated with hand-assisted or totally laparoscopic technique. Another singular feature of our clinical case is the large size: until now the largest neoplasm approached with laparoscopy was 10 cm size [4]. The authors conclude that if it is feasible for retroperitoneal masses laparoscopy ensures a better vision of surgical field, less post-operative pain and better cosmesis. The risks of recurrences associated with an inadequate resection in local invasiveness tumor contraindicate the laparoscopic technique. Prognostic factors are negative margins of resection, histotype and tumor grade as well as tumor integrity in surgical specimen [29]. Horiguchi et al report the appearance of port site metastasis [30]. The feasibility of laparoscopic approach in our case derives from the position of the neoplasm (retroperitoneal space adjacent to the left kidney and left adrenal gland), from the preoperative identification of adiposes cleavage planes with the surrounding peritoneal organs, from the no evidence of invasion of major vascular axes, from the absence of distant metastatic lesions with the possibility to carry out an oncologically radical surgical treatment. A concomitant multivisceral resection is required for adjacent organs involved by the mass or to facilitate the surgical excision of large tumors. A complete in block resection of the neoplastic mass with the left kidney and the ipsilateral adrenal gland derives from the division of retroperitoneum and intraabdominal/pelvic cavity in different compartments, therefore we performed a compartmental surgery demonstrating a reduction in local recurrences [31]. Radical nephrectomy for retroperitoneal liposarcoma adjacent to the kidney showed to increase disease-free survival [32] (no local recurrence is documented in our patient after one-year follow-up).

## 4 Conclusion

A retroperitoneal mass can represent a serious diagnostic challenge and may remain unrecognized until it reaches a large size and this is generally accompanied by a poor prognosis. The high rate of recurrences even after complete resection does not make us able to establish a postoperative follow-up in terms of duration and often it is indefinite. The choice of the best surgical procedure can benefit to the patient prognosis. To our opinion laparoscopy can be a safe and successful treatment respecting the oncological principles and it can represent a valid alternative to open surgery in center with an experienced oncologic and laparoscopic surgical team [[33], [34], [35]]. However we have no randomized controlled trials that compare laparoscopic resection of retroperitoneal liposarcomas with open technique. We can only say that laparoscopy is associated with a shorter hospital stay and less postoperative complications compared to open surgery, but we still have no evidence on the real equality in oncological terms. Therefore it would be necessary to compare the two surgical techniques with clinical trials of at least 5-years follow-up, taking into consideration factors such as tumor recurrence, distant metastasis, length of hospital stay and costs.

## Conflicts of interest

Agrusa Antonino and other co-authors have no conflict of interest.

## Sources of funding

Agrusa Antonino and other co-authors have no study sponsor.

## Ethical approval

Ethical Approval was not necessary for this study.

We obtained written patient consent to publication.

## Consent

We obtained written patient consent to publication.

## Author’s contribution

Agrusa Antonino: study design, data collections, data analysis and writing.

Di Buono Giuseppe: study design, data collections, data analysis and writing.

Buscemi Salvatore: study concept.

Randisi Brenda: data collections, data analysis and writing.

Gulotta Leonardo: data collection.

Sorce Vincenzo: Data collection.

Badalamenti Giuseppe: study design.

Albano Domenico: data collections

Galia Massimo: study design.

Romano Giorgio: study design, data collections, data analysis and writing.

Gulotta Gaspare: study design.

## Registration of research studies

researchregistry4617.

## Guarantor

Agrusa Antonino.

Romano Giorgio.

Gulotta Gaspare.

## Provenance and peer review

Not commissioned, externally peer-reviewed.
